# The challenge of managing ischemic stroke in brucellosis: a case report

**DOI:** 10.3389/fimmu.2024.1347216

**Published:** 2024-03-12

**Authors:** Linfa Chen, Xiaolong Lin, Xiuqu Cai, Shiting Zeng, Yanquan Yuan, Zhiyong Huang, Jinjin Yan, You Li

**Affiliations:** ^1^Department of Neurology, Huizhou Third People’s Hospital, Guangzhou Medical University, Huizhou, China; ^2^Department of Pathology, Huizhou Third People’s Hospital, Guangzhou Medical University, Huizhou, China; ^3^Guangdong Key Laboratory of Age-Related Cardiac and Cerebral Diseases, Affiliated Hospital of Guangdong Medical University, Zhanjiang, China

**Keywords:** brucellosis, stroke, intravenous thrombolysis, T lymphocytes, metagenome next-generation sequencing

## Abstract

A 64-year-old woman was admitted to the hospital for sudden weakness in one of her left limbs. The patient was diagnosed with acute ischemic stroke (IS) of undetermined cause and received intravenous thrombolysis. Following thrombolysis, the patient’s left limb weakness improved, but she subsequently developed recurrent high fever and delirium. Further diagnostic tests revealed that she had been infected with *Brucella melitensis*. The patient showed significant improvement during anti-infection treatment for Brucellosis and secondary prevention treatment for IS. However, her condition unexpectedly worsened on the 44th day after admission due to a hemorrhagic stroke (HS), which required an urgent craniotomy. Immunohistochemical analysis of the hematoma sample collected during the operation showed the presence of CD4^+^ and CD8^+^ T lymphocytes surrounding the blood vessels. This case highlights the unique challenge of managing IS in brucellosis and sheds light on the potential role of T lymphocytes in the immune response related to stroke.

## Introduction

Brucellosis is a zoonotic infectious disease caused by *Brucella*, and it can have various clinical manifestations. However, it is rare for ischemic stroke (IS) to be the initial symptom of brucellosis (1). This article presents a case study of a patient who had brucellosis complicated by IS and hemorrhagic stroke (HS). The objective of this study is to provide clinicians with valuable insights that may assist them in identifying the underlying cause of unexplained strokes and to improve their knowledge and understanding of brucellosis.

## Case presentation

**Figure 1:**
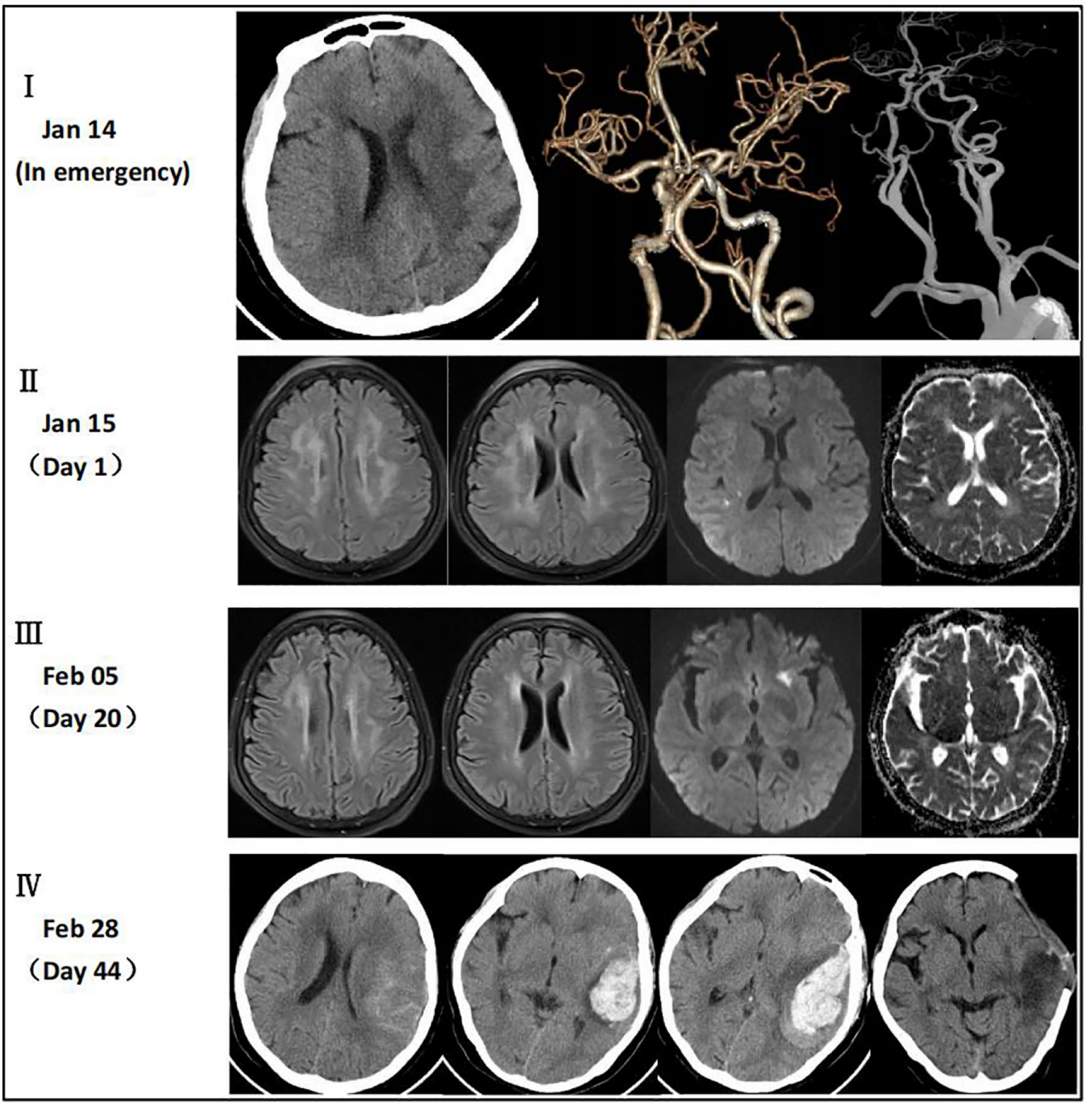
**(I)** CT angiography did not reveal any signs of cerebral hemorrhage or large vessel occlusion. **(II)** MRI showed multiple intracranial demyelination lesions and infarction only in the right temporal lobe and posterior horn of the ventricle. **(III)** MRI showed improvement in the intracranial demyelination lesions and a new IS in the left external capsule. **(IV)** CT scan indicated a HS of approximately 66 mL which was absorbed within 33 days after the operation.

**Figure 2:**
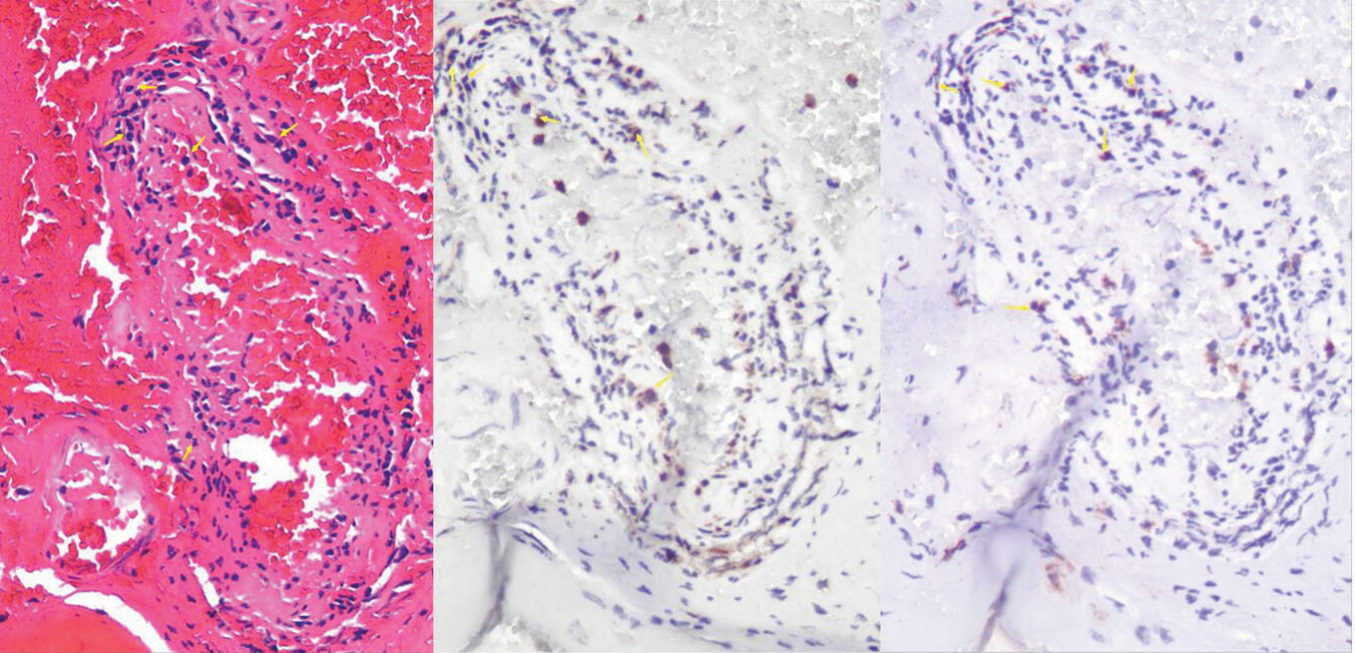
HE staining showed chronic inflammatory cells in the vicinity of the blood vessels. Immunohistochemical analysis demonstrated the infiltration of CD4^+^ and CD8^+^ T lymphocytes around the blood vessels. The yellow arrow indicates the presence of either CD4^+^ or CD8^+^ cells

**Figure 3:**
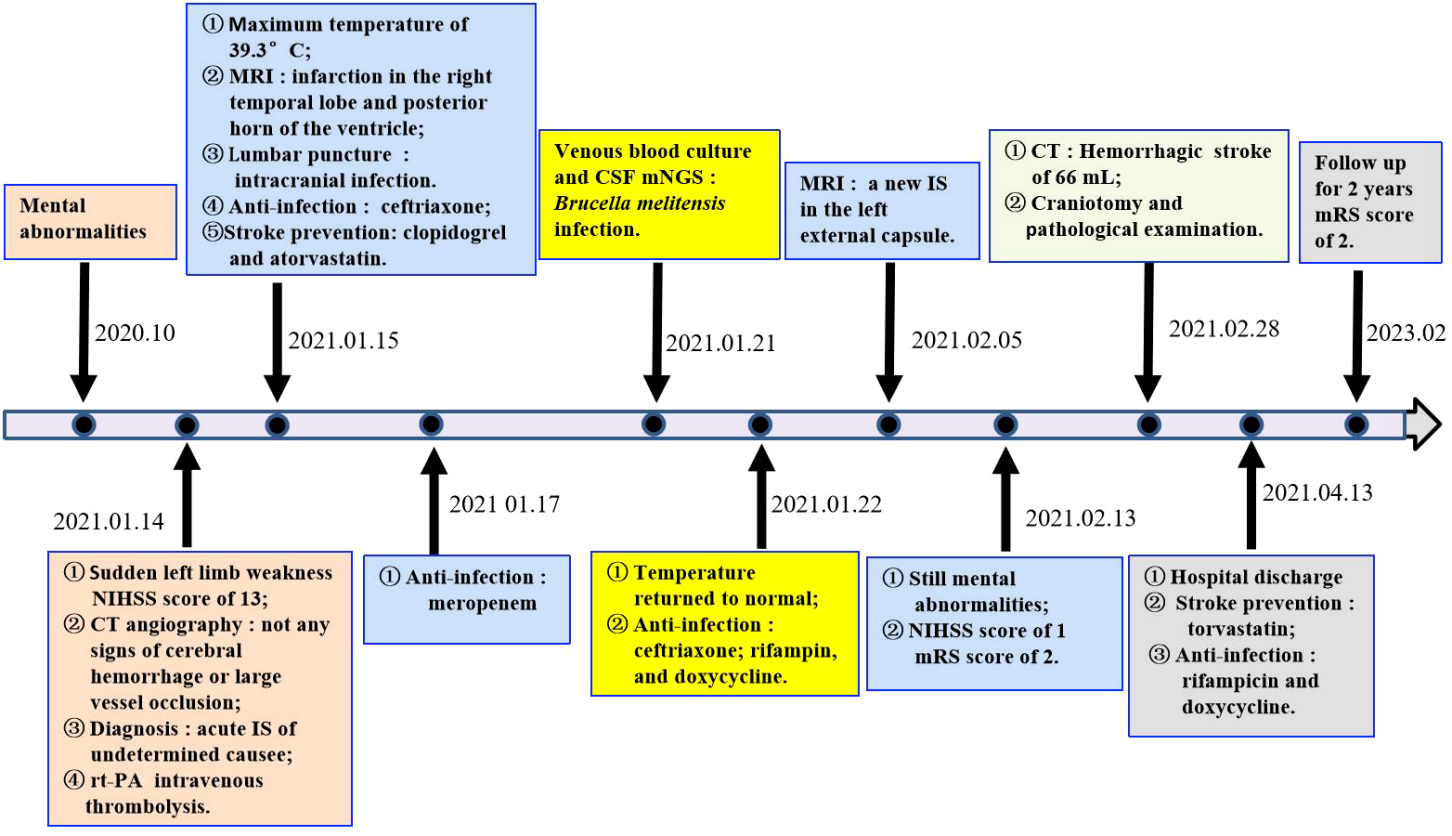
Timeline.

A 64-year-old woman weighing 67 kg who had undergone breast cancer surgery over 10 years earlier presented with mental abnormalities 3 months before coming to the hospital for sudden-onset limb weakness. On January 14, 2021, the patient was admitted to Huizhou Third People’s Hospital due to sudden weakness in her left arm and leg that lasted for 3 h. Physical examination revealed drowsiness, rightward gaze, aphasia, left facial palsy, and left-sided hemiplegia. Her initial National Institute of Health Stroke Scale (NIHSS) score was 13. Head and neck computed tomography(CT) angiography did not show any signs of cerebral hemorrhage or large vessel occlusion (**Figure 1I**). Based on these findings, the patient was diagnosed with acute IS of undetermined cause and received intravenous thrombolysis with recombinant tissue plasminogen activator (rt-PA) 40 mg (0.6 mg/kg) in the emergency room. Following thrombolysis, there was improvement in the patient’s neurological function such that her NIHSS score improved to 8. Brain magnetic resonance imaging(MRI) performed 24 h after thrombolysis showed infarction only in the right temporal lobe and posterior horn of the ventricle (**Figure 1II**). However, the patient developed a high fever 10 h after being transferred to the stroke unit. The patient’s mental function also deteriorated significantly. Lumbar puncture results showed elevated total protein levels (374 mg/dL) and a high nucleated cell count (2.58 × 10^6^/L), along with a decreased cerebrospinal fluid (CSF)/blood glucose ratio. This led us to suspect that the patient had developed IS complicated by an intracranial infection. To treat the infection, we immediately administered ceftriaxone(2 g, q12h), while continuing clopidogrel (CYP2C19 genotype *1/*1) and atorvastatin for secondary prevention. However, even after three days of ceftriaxone treatment, the patient’s fever persisted and the cause of the infection remained unknown. Consequently, on the third day after admission, we discontinued ceftriaxone and transitioned to meropenem (2g q8h) for continued anti-infective therapy. On the 7th day after admission, metagenome next-generation sequencing (mNGS) of CSF and venous blood cultures confirmed infection with *Brucella melitensis.* The new treatment plan comprised intravenous ceftriaxone (2 g, q12h), oral doxycycline (100 mg, bid), and oral rifampicin (0.6 g, qd). Notably, brain MRI conducted on the 20th day after admission revealed a new IS (**Figure 1III**). Subsequently, the patient’s condition worsened. Her Glasgow Coma Scale score was 11 as of 44 days after admission. A brain CT scan indicated a HS of approximately 66 mL, which required an urgent craniotomy (**Figure 1IV**). Immunohistochemical analysis of the hematoma sample collected during the operation revealed CD4^+^ and C8^+^ T lymphocytes surrounding the blood vessels (**Figure 2**). Upon discharge, the patient was prescribed a 3-month regimen of atorvastatin, rifampicin, and doxycycline. The patient attended follow-up appointments at our hospital’s outpatient clinic on the 7th day and 1 month after being discharged. During both visits, normal liver and kidney functions were observed. The blood routine results showed a slightly higher proportion of lymphocytes and monocytes, along with a slightly lower proportion of neutrophils and neutrophil count. Subsequent follow-up calls will occur every 3 months or semi-annually until the end of the follow-up period in February 2023. The patient had regained the ability to walk and had a modified Rankin Scale (mRS) score of 2, although she still experienced incoherent speech (**Figure 3**).

## Discussion

*Brucella* is a facultative intracellular parasite and slow-growing Gram-negative bacillus that can cause acute and chronic damage to various tissues and organs in the human body. Its highly elusive and disabling nature makes it a significant concern. Involvement of the central nervous system in brucellosis is relatively rare, accounting for 0.5%–25% of cases (2). The atypical clinical manifestations of the disease, along with the lack of specific changes in CSF and imaging, contribute to a high prevalence of misdiagnosis and missed diagnosis during the initial stages (3). This case study presents a patient with brucellosis complicated by IS and HS. The aim of this work is to improve clinicians’ awareness and understanding of brucellosis, which may facilitate early diagnosis and prompt treatment and so reduce patient morbidity and mortality.

The mechanisms by which *Brucella* evades the host immune response are not fully understood. Previous research has suggested that *Brucella* enters the human body through damaged skin, mucous membranes, or the digestive tract. It is believed that *Brucella* is initially engulfed by macrophages and dendritic cells, where it grows and multiplies, leading to the formation of infectious lesions. When *Brucella* breaches host cells and re-enters the bloodstream, it continues to grow and multiply in various tissues and organs, causing bacteremia and clinical emergencies (4). Currently, there is a growing interest in studying immune protection mechanisms involving CD4^+^ and CD8^+^ T lymphocytes after *Brucella* infection (5). Recent studies have found that patients with brucellosis have a reduced proportion of CD4^+^ T lymphocytes in their peripheral blood but an increased proportion of CD8^+^ T lymphocytes (6). Some studies have suggested that *Brucella* infection disrupts the interaction between B lymphocytes and CD4^+^ T lymphocytes, thereby increasing the host’s susceptibility to *Brucella* (7). Hematoxylin and eosin (HE) staining showed a notable presence of chronic inflammatory cells in the vicinity of the blood vessels (**Figure 2**). Immunohistochemical analysis indicated the infiltration of CD4^+^ and CD8^+^ T lymphocytes around the blood vessels, which is consistent with previous findings (8, 9). Our hypothesis is that T lymphocytes may be involved in chronic vascular immune inflammation associated with stroke. Recent studies have provided evidence for this hypothesis and emphasized the important role of lymphocytes in reducing local complications of *Brucella* infection (10). Another study has shown that a live mucosal vaccine can reduce the damage caused by *Brucella melitensis* through CD4^+^ and CD8^+^ T lymphocyte immunity (11). Nevertheless, there have been a few reported cases of brucellosis complicated by HS. Previous studies indicate the involvement of infiltrating T lymphocytes in the pathophysiological process of cerebral hemorrhage (12). However, the precise mechanism by which brucellosis contributes to the occurrence and progression of stroke requires further investigation.

The diagnosis of *Brucella* infection can be confirmed through culture, serological testing, or nucleic acid amplification tests. Previous studies have shown that CSF and venous blood cultures have a low positive rate and are time consuming (13). However, with the advancement of automated blood culture systems, *Brucella* can now be detected in over 95% of blood cultures collected from patients with acute brucellosis within 1 week (14). In this case, we had inquired about the patient’s medical history and family history, but neither the patient nor his family members could provide information regarding possible pathogenic microbial infection and the route of infection. This lack of information posed challenges for clinical diagnosis. Fortunately, when the patient had an elevated temperature prior to antibiotics, we conducted a venous blood culture in time to confirm the *Brucella* infection. We also performed two enhanced brain MR scans and identified multiple intracranial demyelinating lesions. It has been reported in previous literature that the brain MR White matter demyelination of neurobrucellosis is related to T lymphocyte immune response (8). Although the results lacked diagnostic specificity, we discovered that the patient had experienced two IS events. This case report highlights the significance of conducting a brain MR examination in patients with neurological brucellosis. It not only aids in differential diagnosis but also enables timely detection of IS and facilitates the administration of secondary prevention treatment. Furthermore, mNGS has gained widespread acknowledgment as a valuable tool for its high-throughput, rapid, and accurate diagnosis of unexplained intracranial infections (15). Consequently, we leveraged mNGS to analyze the CSF for pathogenic microorganisms, resulting in the identification of the pathogen *Brucella melitensis*.

The treatment of brucellosis primarily focuses on implementing anti-infection measures. However, there is currently no standardized approach for managing complications. In this case, rt-PA was administered, which showed a noticeable therapeutic effect. There are no previous reports of brucellosis patients with IS receiving intravenous thrombolysis. Despite initiating clopidogrel and (75 mg/day) and atorvastatin (40 mg/day) as secondary prevention treatment for stroke, the patient still experienced a new IS and HS. Although the patient’s intracranial demyelination lesions improved with anti-infection therapy, her psychotic symptoms did not improve significantly. Therefore, the use of glucocorticoids should be considered because they have some established efficacy in treating neurological brucellosis (16). However, further robust evidence is required to support this claim.

## Conclusion

This study presents the first evidence of chronic inflammatory cells surrounding the blood vessels in cases of brucellosis complicated by HS. The presence of CD4^+^ and CD8^+^ T lymphocytes around the blood vessels in brucellosis patients provides a clinical basis for future research on the mechanisms underlying stroke in brucellosis complications. In cases of IS of clinically undetermined cause, intravenous thrombolytic therapy may alleviate the patient’s clinical symptoms. To improve the clinical diagnosis of IS and ensure timely secondary prevention of stroke, it is recommended that patients with *Brucella* infection undergo brain MR examination. Additionally, venous blood culture remains a reliable method for clinically diagnosing pathogens, and mNGS can quickly identify pathogenic organisms in CSF.

## Data availability statement

The original contributions presented in the study are included in the article/supplementary material. Further inquiries can be directed to the corresponding authors.

## Ethics statement

The studies involving humans were approved by Huizhou Third People’s Hospital of Guangzhou Medical University. The studies were conducted in accordance with the local legislation and institutional requirements. The participants provided their written informed consent to participate in this study. Written informed consent was obtained from the individual(s) for the publication of any potentially identifiable images or data included in this article. Written informed consent was obtained from the participant/patient(s) for the publication of this case report.

## Author contributions

LC: Data curation, Formal analysis, Funding acquisition, Investigation, Writing – original draft, Resources, Visualization, Writing – review & editing. XL: Writing – review & editing, Data curation, Resources. XC: Data curation, Writing – review & editing. SZ: Formal analysis, Investigation, Writing – review & editing. YY: Formal analysis, Writing – review & editing. ZH: Project administration, Resources, Visualization, Writing – review & editing. JY: Project administration, Resources, Visualization, Writing – review & editing. YL: Conceptualization, Formal analysis, Project administration, Supervision, Visualization, Writing – original draft, Writing – review & editing.
